# Bridging the gap: investigating challenges and way forward for intersectoral provision of psychosocial rehabilitation in South Africa

**DOI:** 10.1186/s13033-016-0042-1

**Published:** 2016-03-09

**Authors:** Carrie Brooke-Sumner, Crick Lund, Inge Petersen

**Affiliations:** School of Applied Human Sciences, Discipline of Psychology, University of KwaZulu-Natal, Durban, South Africa; Department of Psychiatry and Mental Health, Alan J Flisher Centre for Public Mental Health, University of Cape Town, Cape Town, South Africa

**Keywords:** Intersectoral collaboration, Partnerships, Psychosocial rehabilitation, Severe mental illness, Community-based rehabilitation, Community development, Mental health policy

## Abstract

**Background:**

Intersectoral collaboration between government sectors such as Health and Social Development and non-governmental organisations (NGOs) in communities is crucial for provision of psychosocial rehabilitation (PSR) for those with severe mental illness. This study aims to provide recommendations for strengthening such intersectoral collaboration in South Africa and with relevance to other low and middle income countries (LMIC), particularly African countries.

**Methods:**

Twenty-four in-depth semi-structured interviews were conducted with 16 key informants from the South African Department of Health, two key informants from the Department of Social Development, four key informants from the NGO sector and one key informant from a service user organisation at national level. Framework analysis was conducted with NVivo 10 software.

**Results:**

Challenges to intersectoral work identified were lack of communication between sectors, problems delineating roles, and each sector’s perception of lack of support from other sectors. Participant-identified strategies for addressing these challenges included improving communication between sectors, promoting leadership from all levels and formalising intersectoral relationships through appropriate written agreements; as well as ensuring that the available resources for PSR are effectively re-directed to district level.

**Conclusions:**

This study has outlined several directions for progress to address challenges for intersectoral working for PSR in South Africa. These may be of relevance to other LMIC, particularly those in Africa. Political will and a long-term view will be necessary to realise these strategies.

## Background

WHO defines ‘intersectoral action for health’ as a relationship between the health sector and other sectors which is necessary to improve health outcomes more effectively, efficiently or sustainably than would be achieved by the sole action of the health sector [1]. The need for intersectoral collaboration in the provision of comprehensive community-based mental health services is well recognised internationally and in South Africa [2–7]. The WHO Mental Health Action Plan 2013–2020 also cites a main objective of improving provision of integrated mental health and social care services in communities [8].

Intersectoral collaboration is crucial, particularly for provision of psychosocial rehabilitation (PSR) for those with severe mental illness (a mental disorder meeting DSM5 and/or ICD10 diagnostic criteria and causing serious functional impairment). These individuals are recognized as having a range of medical and psychosocial needs [9] whether they are hospitalised or living in the community. The potential benefits of an intersectoral approach are well accepted and in high income countries (HIC) intersectoral work is mandated for a range of health and social services [10]. For example, models of Assertive Community Treatment and more recently Intensive Case Management involve multidisciplinary specialist community-based teams (comprising psychiatrists, nurses, social workers, psychologists, occupational therapists and others) [11, 12] and rely on partnerships with service users, families and local community services, including social welfare and housing sectors [13]. The human resource crisis for mental health in many low and middle income countries (LMIC) currently precludes the feasibility of a community-based specialist team for PSR. Provision of adequate long-term care in the community in LMIC, within the real-world resource-constrained context, will however of necessity require collaboration between the relevant government and non-government sectors.

In the African context although some countries have policies on development of community-based services, actual implementation of these policies has proved challenging [14]. One reason for this in South Africa [15], as internationally [16], is rapid progress in down-sizing of specialist psychiatric hospitals that has not been accompanied by the recommended ring-fencing of money saved for direction to community-based services [2, 4]. As in some HICs in the past, the process of deinstitutionalisation in South Africa in particular has been viewed as an opportunity to cut mental health budgets [7] and the overall low level of resources for psychosocial community-based services persists [2, 17–19]. This challenge may be compounded by lack of skills on the part of managers and those implementing policy to advocate for resource allocation for community-based services in the milieu of competing health and mental health priorities [9], a challenge that may not be limited to South Africa or other LMIC settings.

Similar to the situation in other LMIC, there are therefore important gaps in the provision of PSR services in South Africa, particularly in rural areas [2]. The Government Health and Social Development sectors are clearly mandated in national policy for provision of PSR. Current levels of service provision however vary widely across provinces, with the National Department of Health (DOH) remaining focused on a biomedical treatment model and the National Department of Social Development (DOSD) activities being limited to provision of disability grants and funding of non-governmental organisations (NGOs). As in other LMIC, current service provision for PSR in South Africa thus continues to be mainly through NGOs [7] (e.g. South African Federation for Mental Health). These NGOs are partially funded by DOSD to provide this service, under the DOSD Policy on Disability [20]. Since DOH is not routinely funding NGOs in a similar way for provision of PSR services, the question remains as to what extent savings resulting from deinstitutionalisation are following patients into the community [7, 21]. Experience of PSR in LMIC indicates NGOs are typically limited in their ability to provide sustainable services. NGOs in middle income countries particularly may find it challenging to secure sustainable donor funding. The failure to fully integrate NGO services with those provided by government Health and Welfare sectors to ensure continuity of care and provision of the full range of services required also challenges sustainability and limits the quality of care provided [22].

Addressing the PSR service gap is a key challenge in South Africa [23] and other LMIC. Notably in South Africa this service is urgently needed to reduce the revolving door phenomenon (repeated discharge into the community followed by rehospitalisation) and high numbers of individuals with severe mental illness being homeless or living in prisons [15]. Within the health sector in South Africa, there is limited care for those with severe mental illness in primary care except for symptom management through the provision of ongoing antipsychotic medication. Lack of capacity at this level for medication management, poor links with other levels of the health system and supply chain issues are however known to lead to inconsistency in the availability of medications and reduced adherence [24, 25]. Time constraints on clinic staff lead to nurses providing a service of dispensing medication with little psychosocial intervention [11]. Furthermore a lack of orientation and skills of service providers towards holistic and chronic care is also an issue, although DOH is making inroads into addressing this [26]. DOH has in fact made important progress towards the provision of comprehensive mental health services, particularly through the development of the National Mental Health Policy Framework and Strategic Plan 2013–2020 [15]. While this includes provision for community residential care and day care services as well as task-shared community-based rehabilitation programmes (PSR) in all provinces, implementation remains a challenge. There is however growing evidence from LMIC on task-shared interventions for PSR (e.g. [27]) and a recent study showed potential for task-shared PSR in low-resource South African settings [28]. The National Mental Health Policy highlights the role of intersectoral collaboration between Departments of Education, Social Development, Labour, Criminal Justice, Human Settlements and NGOs. Some progress is noted to have been made on intersectoral collaboration at the national level but at the provincial and district levels such collaboration is rare [15]. A key provision of this policy is the establishment of specialist mental health teams at district level which will have responsibility for operationalisation of the framework and have important potential to move forward progress on intersectoral collaboration at the district level.

This study aims to document perspectives of a range of key informants regarding current challenges and the way forward for intersectoral provision of PSR. This was done with a view to providing recommendations for strengthening intersectoral collaboration. These insights may be of use to other middle income countries contending with the complexity of intersectoral working, as well as to low income countries, particularly in Africa, which may have different policy and service delivery contexts, but which could benefit from a strengthened intersectoral approach as mental health services develop.

## Methods

### Study context

This study is a subcomponent of The PRogramme for Improving Mental health carE (PRIME), a research consortium implementing interventions for priority mental disorders in low-resource settings [29]. PRIME in South Africa conducted a situation analysis which showed limited provision of community-based PSR [30] in the PRIME study district, Dr. Kenneth Kaunda District, North West Province [31]. PRIME is also implementing a district mental health plan, incorporating a collaborative care PSR component for people with schizophrenia reported elsewhere [28, 31, 32].

### Design

An in-depth qualitative approach was employed to investigate perspectives of key informants. Semi-structured in-depth interviews were used to generate qualitative data reported here based on the consolidated criteria for reporting qualitative research (COREQ) checklist guidance [33]. The framework method of analysis was used, which is regarded as suitable for distilling recommendations for guiding health systems and policy development [34]. Advantages of this approach for this study include its suitability for use for individual interview data from different types of participants and the ability to easily compare responses between participants using a framework matrix [35].

### Sample and procedure

Purposive sampling was used to recruit key informants (24 in total) comprising managers and policy makers at national, provincial and district levels. Twenty-four in-depth semi-structured interviews were conducted with 16 key informants from DOH (three national level representatives (policy makers), four provincial level representatives (health programme planners), nine district level representatives (primary health care managers, nurses, mental health coordinator); two key informants from the DOSD (one national level representative, one provincial level representative); four key informants from the NGO the South African Federation for Mental Health (one national level representative, three district level representatives) as well as one key informant from a service user organisation at national level. Interviews were conducted in person or telephonically. Interviews were conducted in English by the first author and an MPsych graduate, lasted between 45 min and 1 h, and were recorded and transcribed verbatim, with the participants’ consent.

### Analysis

NVivo10 data analysis software was used to store data and help conduct framework analysis. The process of framework analysis, conducted by the first author, incorporated familiarisation with the data through review, application of the framework to enable initial coding, identification of subthemes within the framework through inductive coding and refining of codes and themes [34–36] until no new themes emerged. Major themes of ‘Views on current levels of intersectoral collaboration’, ‘Challenges to intersectoral collaboration’ and ‘Strategies for addressing challenges’ formed the initial coding framework, with additional themes being added as coding of data took place. The data analysis, while employing a framework, did allow for flexibility and emergent themes throughout.

### Ethical considerations

Ethical approval for the study was granted by the Biomedical Research Ethics Committee (BREC) at the University of KwaZulu-Natal (approval numbers BE407/13; HSS/0623/012D) and the University of Cape Town (UCT HREC 412/2011). Participants were informed as to the aims and scope of the interviews and as to the voluntary nature of their participation. Informed consent was obtained from all participants. Anonymity of respondents’ data was ensured throughout analysis and writing up through allocation of identifier codes. Interviews were stored on password-protected computers.

## Results

### Theme 1: views on current levels of intersectoral collaboration

Participants from DOH at district (six) and provincial (three) level, as well as the DOSD provincial representative and NGO (two district, one national) and the service user representatives agreed that existing levels of intersectoral collaboration were inadequate, with two stating that collaboration was ‘almost zero’ and that there was ‘no collaboration’ at district level. However, several participants did describe examples of intersectoral collaboration directed by the initiatives of individual staff and relationships built between sectors in an unstructured way. A DOSD provincial representative cited a case in which a person with schizophrenia was identified by the DOSD provincial office for participation in a Department of Public Works employment programme for people with disabilities. The service user organisation representative (based in Gauteng province) described the role of the DOH mental health coordinator who visited their community residential facility in strengthening relationships between DOH and NGOs.

*‘I think she [DOH mental health coordinator] has monthly meetings with representatives from all the NGOs…and she has developed quite an open communication. She does visit the [community residential] centre now and again and what I like is that she speaks to the residents… to check that they are happy with the service they receive.’*—Service user representative 1

### Theme 2: current challenges to intersectoral collaboration

Participants identified three main challenges, reported here as subthemes.

#### Subtheme 1: lack of communication and structured working relationship

Participants from all sectors and all levels cited lack of structured relationships and effective communication as a major barrier to intersectoral work. At district level, a DOH representative described challenges in referring and following up mental health service users to DOSD since social workers were based in the district office not in the community and there was no back-referral or established communication mechanism.

*‘It’s a challenge because of the communication channel that we have been told to use to access the social worker. You are told that you have to write a letter and explain your problem and send it over to Social Development. We don’t know who is actually receiving it and which channels is it going through. We don’t even get feedback if the social worker was there.’*—DOH District representative 10

A DOH provincial representative identified the challenge of people within DOH and other sectors working in ‘silos’ focusing on their objectives and personal recognition. A district level NGO representative described individualised cases where communication with DOH was functional through specific relationships with clinic staff, but described a major challenge to their work due to lack of information (due to confidentiality concerns) provided to NGOs on diagnosis of patients being down-referred from psychiatric hospitals. The National NGO representative corroborated this and made the link between the lack of communication and lack of service delivery and holistic treatment of clients.

*‘I think there needs to be a proper structure. At this stage there is no communication. And lack of communication actually has a detrimental effect of no service delivery.’*—NGO National representative 1

DOH, DOSD and NGO representatives described the lack of a functioning coordination forum to enable communication. One district level DOH representative described a forum that had been set up but which experienced challenges in that representatives would fail to attend the meeting due to conflicting priorities. By contrast, a district level NGO representative described being part of a forum on disability, which did not have a representative from DOH. Two DOH and one DOSD provincial representative noted the lack of communication and working structure at district and lower levels, despite the Social Development ‘cluster’ (Departments of Health, social development, education, children and people with disabilities) being present at provincial level.

*‘At provincial levels, the departments are arranged into clusters, so there’s the Social Development cluster …. And I’ve sat at those levels [district, ward], and the practical integration is just not there at all. If that was to be effective, that same structure [Social Development cluster] needs to be in place at district level, at a sub*-*district level and at a ward level.’*—DOH Provincial representative 1

#### Subtheme 2: problem delineating roles

The majority of participants described ongoing lack of clarity as to the roles of the different sectors in PSR. Respondents had varying opinions as to the role to be played by sectors other than their own and overall the respondents from DOH and NGO sectors felt that DOSD inadequately fulfilled its role. This was exemplified in the provision of community-based residential facilities for people with severe mental illness. A DOH national representative noted that this requires a ‘number of inputs’ from Housing and Social Development in particular and the inter-dependence of inputs from the different sectors for a comprehensive service was seen to further complicate the relationship between sectors.

*‘But when it comes to psychosocial rehabilitation, it becomes a bit tricky because, for one to provide psychosocial rehabilitation and community*-*based residential services, there are a number of inputs….for a district to establish a community residential facility, we would need capital funding for the structure. And currently, Social Development is saying, we are not the ones to provide a structure. We will just provide the rehab services, we’ll provide social grants, you know. So it becomes complex for the Department of Health to implement community based services without the other parties also bringing their input on the table.’*—DOH national representative 3

A DOH provincial representative felt that provision and management of community residential services is not recognised as a health ‘competency’, leading to the ongoing question as to whether DOH or DOSD should manage these facilities. By contrast, a DOH national representative noted that mental health care users access services initially through hospitals and clinics, creating the expectation from DOSD that DOH should bear responsibility for ongoing support.

*‘If we look at mental health care users, Health is the one that is serving the users. So, the possibility is that… will the Department of Social Development really support what their policies say around provision of residential facilities?.’*—DOH national representative 3

Another DOH representative acknowledged that social workers and occupational therapists were key to provision of PSR, and while these cadres are employed by DOH he questioned whether their numbers are sufficient and whether they were accessible to mental health users residing in the community.

From the DOSD perspective, nationally the main challenge seemed to be the lack of a clear strategy that outlines the role to be played by DOSD. A national level DOH representative felt that the main role of DOSD is provision of disability grants, and acknowledged that the partial funding by this department to NGOs providing mental health services may be inadequate. DOSD representatives were clear on their framework for action, with NGOs funded by DOSD representing their ‘implementation arm’ in a service delivery model enabling local organisations to respond to needs in their community.

From the NGO perspective, the national representative recognised their role in provision of community services with DOSD funding, but thought that the focus of DOH in providing for needs in a biomedical model (diagnosis and medication) with a lack of focus on psychosocial support, and the corresponding reluctance of DOH to hire social workers (seeing this as mandate of Social Development) led to a failure in continuity of care.

*‘Health is very good at saying ‘we will train foot soldiers, volunteers and home care workers’, but they will not employ social workers because that lies with Social Development.’*—NGO National representative 1

A NGO district representative also felt that DOSD could have a more active role in providing services (e.g. support groups and awareness campaigns).

Representatives from DOH, DOSD and the NGO national representative all felt that the lack of a service level agreement meant that it was difficult to ensure provision for sector responsibilities. The DOH and DOSD representatives drew the comparison to the substance abuse plan in which sectors’ roles are elaborated and clarified in signed agreements. Despite lack of clarity on specific roles, participants from the DOH, DOSD and NGO sectors broadly agreed that ‘leadership’ on provision of PSR services and resourcing should come from DOH. One DOH national representative cited the need for health to lead on the reorganisation of services to ensure resources spent on specialist facilities filter to community care.

*‘So we’re going to organise the system, we’re going to improve the system, so we take the leadership … if there are things that are not being done, we have to do them. Health must stimulate that work.’*—DOH National representative 2

DOH and DOSD representatives acknowledged that the DOSD model of funding NGOs in communities to provide services was an appropriate strategy for delivery of community-based services.

#### Subtheme 3: perceived lack of support from other sectors

Representatives from all sectors held perceptions of lack of support and trust in other sectors to fulfil their roles. Several DOH representatives described challenges partnering with DOSD. District DOH representatives described challenges in accessing DOSD social workers assigned to their health facilities, acknowledging that social workers seemed overburdened.

*‘They [social workers] have wards where they work in, but the minute we ask them to go to a certain address then they say, no it’s not my area… that makes it quite difficult for us.’*—DOH District representative 3

However it was acknowledged by a DOH national representative that there is strong support at national level from DOSD in terms of the overall community development approach within in which PSR is located.

*‘If you find gaps [in PSR], your remedies are largely around developing a community … so that’s development work which largely Social Development is well*-*skilled in facilitating. So I know that my colleague [in DOSD] has the interaction at national level to align the policies and they agree on what needs to be done.’*—DOH National representative 2

An NGO district representative described the ongoing struggle to access funding from DOSD.

*‘Interviewer: So the service needs to be set up and running before?*

*Respondent: Yes, before they will give us any funding. And the thing is that, we have no assurance that if we start rendering the service this month next month we will receive subsidy. There are some organisations that have been rendering services for long years not receiving subsidy and we do not have the funds to do that.’*—NGO District representative 1

The service user representative perceived a lack of support from DOSD in their failure to provide for the specific need for psychologists in community residential facilities and to provide collaboration and support necessary in providing the service.

*‘I think they [DOS] are too little involved. …They are not too actively involved in kind of partnering and saying let us look at more effective ways… let us look at a bit of research on psychosocial rehabilitation, what models work best. You know, kind of that collaboration and support. I am not talking only about funding, but other support.’*—Service user representative 1

From the DOSD perspective, a Provincial DOSD representative described difficulties in working with DOH as a partner and particularly a lack of joint working between DOSD disability coordinators and health staff in facilities, and on raising awareness around psychosocial disability (i.e. the impaired social and role functioning caused by mental illnesses).

### Theme 3: Strategies for addressing challenges

Participants identified three major strategies to address the above challenges, reported here as subthemes.

#### Subtheme 1: promoting clarity on roles

Participants generally agreed on the need for roles of sectors and staff to be clarified. Respondents from DOH district and national levels emphasized that role clarification would enable better care specifically by allowing more efficient referrals between the sectors (e.g. between DOH and NGOs in the community).

*‘…role verification for each of the departments [is needed], so that they know exactly what is their role, and how they can assist. Say for instance an NGO like Mental Health [Society], they are so knowledgeable, whereas maybe the skills of professional nurses on how to manage a client on community level are maybe limited.’*—DOH District representative 4

In terms of the specifics of the roles each sector should be playing, participants described activities that reflect the current activities of the different sectors, with the suggestion being that performance and fulfilment of roles be strengthened.

#### Role of DOH

Participants agreed that DOH should take the lead role in improving the integrated care of people with severe mental illness through national to provincial and district coordination of the intersectoral provision of services. Three DOH national representatives described a need for strengthening capacity at provincial level to enable health service planners and managers to effectively conduct integrated planning with other departments.

*‘… basically our view is that if we can strengthen the capacity for the provincial offices to consult, develop plans together, find the other partners who need to be part of it [PSR] ….we should be able to stimulate this kind of work and ensure that intersectoral collaboration takes place’*—DOH national representative 2

District DOH representatives and the NGO national representative highlighted the need for a strengthened role of DOH staff in providing psychoeducation for service users and families (health education on the mental illness and its management). District level DOH representatives cited a need for an increased role of community health workers in encouraging the involvement of community members and family in ongoing support and referring mental health users to support groups. A specific role of specialist mental health teams laid out in the new Mental Health policy was also articulated by a DOH national representative, in that they should be responsible for creating and maintaining a plan covering gaps in current service provision and setting up mechanisms for intersectoral collaboration at the district level.

*‘And in the terms of reference of that district mental health team, this is one of the areas that we listed*—*that they should be able to produce a plan for a district. For instance, look at where are the gaps in these areas, create a structure … create mechanisms of collaborating with the other key sectors, whether it’s Education, Social Development and all that.’*—DOH national representative 2

District and provincial DOH representatives also described the need for community-based DOH-employed social workers (as part of specialist mental health teams) and acknowledged that additional numbers of social workers would need to be employed by DOH.

*‘I know that in other places they have medical social workers at the hospitals but not at primary health care. We don’t have a social worker. Again, in most cases we find that the psychologist will then report to you that most of the cases need social workers intervention and therefore it becomes a challenge with referral and also giving feedback to other departments.’*—DOH District representative 2

#### Role of DOSD

DOH district participants identified key roles of DOSD in the provision of disability grants through SASSA. DOH and DOSD national representatives felt the main role of DOSD going forward was to strengthen the current mode of service provision through funding a wider network of NGOs. Beyond this, DOSD was also identified in having a role in an overall community development approach encouraging ‘informal services’ and support in the community through empowering families and community members to better address the needs in their community

*‘So Social Development, if we are to give effect to the development of community*-*based mental health services… need to embrace the user organisations, ensure that they work with families and encourage, beyond what government can do, also encourage what we call the development of informal services. Because, look, I think we appreciate that, yes, there’s a lot of government should facilitate, or the two departments [Health, Social Development] should do, but some of the good innovations can be found when you encourage communities informally to address their needs. It’s a lot of development work.’*—DOH national representative 2

While DOH and DOSD participants acknowledged that DOSD social workers were overwhelmed with other social care needs, DOH district representatives also held the perspective that they needed to find a way to work in conjunction with social workers from planned specialist mental health teams. They also highlighted the importance of a functioning referral system between health facilities (including specialist mental health teams) and DOSD social workers.

The DOSD provincial representative felt that DOSD social workers did have more of a role to play in addressing the significant strain on families of mental health service users. This participant also suggested the need for a mental health specialisation for DOSD social workers, similar to the current specialisation in children’s services. The NGO national representative suggested that DOSD should be placing a departmental social worker in all primary health facilities, which was the case in some areas but not standardised across the provinces.

*‘But that would be a recommendation, that while Health provides at a primary setting a nurse, and access to a psychiatrist, Social Development should be providing a social worker at that level.’*—NGO national representative 1

#### Role of NGOs

Discussion on the role of NGOs centred on the Mental Health Societies (provincial arms of the South African Federation for Mental Health) as these are the main NGOs currently providing services. Representatives from all sectors agreed that NGOs were best placed to provide PSR services on the ground. The NGO national representative agreed with this perspective but highlighted the need for adequate resources to be directed to these services.

*‘…so it is the state’s responsibility to make sure the structures and the resources are in place. It is wonderful if civil society is used because that’s where the skills lie, in terms of civil society providing a service which government needs to purchase. But it has to be a valuable service, so not a totally cheap service where you compromise on the service delivery.’*—NGO national representative 1

DOH district and national participants suggested a need for a wider network of NGOs providing support services. They also identified key roles of NGOs in managing residential facilities, providing for productive or income generating activities and addressing ‘general social needs’. The DOSD national representative held the perception that NGOs also have a key role to play in empowering mental health service users to be involved themselves in service provision.

*‘…you need to actually make sure that the NGOs are also consulted … because we look at empowering mental health care users, or people with mental disabilities to get employed or run businesses, or render services. What needs to actually happen is because of the scarcity of resources … we then can look into capacitating people with disabilities themselves, and the family members who are passionate or understand this kind of illness or disability, to be part of the service delivery at provincial and local level.’*—DOSD national representative 1

#### Need for a coordination role

Participants involved in the direct provision of services strongly emphasized the need for a focal person who would coordinate PSR services and collaboration between sectors. DOH representatives at district level identified the person to fulfil this role as the DOH district mental health coordinator [a function that is only present in some districts and is likely to be replaced by specialist mental health teams, in keeping with the new national Mental Health Policy Framework (2013–2020)]. Specific roles for this coordination function included liaising with intersectoral partners (service providers at management level), community members and ward counsellors, taking up PSR issues at social cluster meetings, and contributing to financial and operational planning for PSR services.

#### Need for a case management role

Participants also identified the need for a focal person who would fulfil the case management role for individual patients. This included follow up of patients in the community, working with families, liaising with hospital staff and other service providers on referrals, and following up treatment defaulters. NGO participants, those involved in current provision of these services, felt this should be a social worker.

*‘…social workers play the key role of coordination, of making sure there is follow up, making sure the family support structures are in place and other support structures. So it’s the community development part where social workers are involved’*—NGO national representative 1

#### Subtheme 2: improving communication and structured working relationship

Participants identified four strategies for improving the communication and working relationship between the sectors.

#### Leadership

DOH representatives from district, provincial and national levels called for leadership in intersectoral collaboration from DOH. Participants from the different levels of service delivery had differing perspectives on how this should take place. Some district and provincial level participants felt that leadership should come from high levels of the health system.

#### Role of NGOs

*‘Whatever comes from the political side and is emphasized by politicians has more value because the people listen to politicians. If we can have politicians emphasising the importance of support from the community and also improving services, then to some extent we can improve the services.’*—DOH district representative 9

One provincial DOH representative also emphasized the need for leadership at all levels:

*‘We are envisaging for this kind of collaboration to take place at all levels, whereby even the executive managers meet and talk about particular issues … so that when the implementers come in, it’s not about them having to pave the way forward for how they are going to work.’*—DOH provincial representative 4

#### Formalised relationships

DOSD national and provincial representatives specifically noted the need for a memorandum of understanding to provide a grounding for the working relationship between DOH and DOSD. While only one DOH district representative articulated the need for a service level agreement, other DOH participants noted the lack of structure to the intersectoral relationship.

*‘I personally believe there should be something like a memorandum of understanding between the departments, in order to enforce a formal relationship in terms of service provision.’*—DOSD Provincial representative 1

#### Improving communication and referrals between sectors

The need for regular intersectoral meetings was expressed by DOH and NGO district representatives. Similarly a DOH provincial representative suggested the need for closer working relationships with DOSD counterparts including daily communication.

*‘.Maybe just getting the system in place where there are regular meetings to say this is what we struggle with…because there are no meetings between health and NGO’s and social development focusing only on people with disability.’*—NGO District representative 4

Four DOH district representatives identified the existing sub-district Social Development Cluster meetings as a crucial opportunity for intersectoral communication, while one acknowledged that mental health had not been a priority in these meetings and another questioned the effectiveness of the structure. One made the suggestion that ward councillors also be involved in these meetings and another suggested the need to involve traditional healers.

*‘Each sub*-*district has a Social [Development] Cluster meeting with the different intersectoral partners, and that’s where there should be some integration. It’s not very functional, what happens is that … like Social Development will send one person this week and then next month another person so there’s no continuity, so the same issues come out at every meeting and nothing really moves. But that would be where it should happen, but it’s not happening.’*—DOH District representative 8

Participants from all sectors described the need to improve referrals between the sectors. Specifically the need for an effective referral and feedback pathway between DOH, NGOs and DOSD social workers was highlighted by a DOH district representative and the NGO national representative. Social workers were seen as providing the crucial link from health services to other services and resources in the community.

*‘Once the person has seen the psychiatrist and is on medication where do you refer to? And that referral must be a proper one, you can’t just refer to a social work department or the Department of Social Development…that task team should be in contact with one another all the time.’*—NGO national representative 1

In line with this, a DOH national representative highlighted that a key role of the specialist mental health teams outlined in the Mental Health Strategy will be in clarifying referral pathways and ensuring continuity of care.

#### Subtheme 3: appropriate resource allocation for PSR

Contrasting views between national and district level participants on allocation of resources for PSR emerged. DOH and NGO representatives at district level felt strongly that additional resources should be allocated to their organisations or to community mental health services.

*‘For example we have an HIV budget and HIV funds that are continually announced at budget speeches … if this service for mental health [PSR] can be integrated it will be a great improvement to our services because then it means you don’t have to struggle to get resources.’*—DOH district representative 9.

However DOH national representatives and the NGO national representative felt that resources for PSR were available taking into consideration resources between the sectors, although currently not specifically allocated to PSR. One DOH national representative felt that the likelihood of getting more resources for mental health is low at this point so what is needed is an analysis of services provided and reorganisation of budgets to enable more efficient use of resources. Another highlighted the need for PSR services to be defined in district mental health budgets for community-based services, indicating that the budgeting process in this regard needs improvement across provinces.

*‘Because service delivery is within Social Development, within a specific subdirectorate, and then also within Health, it is very isolated. And these are at national structures, but when it goes down to the provincial I don’t think that… in terms of allocation of funding… it’s not stipulated that within mental health this is the service package. I think that the biggest issue is the lack of interdepartmental cooperation, so there is budget allocated to different departments and if this is utilized correctly then these resources are there to be used.’*—NGO National representative 1.

#### Provision of infrastructure

DOH representatives from district, provincial and national as well as the service user representative emphasized that a priority for future intersectoral work should be the provision of infrastructure. This was seen to be both through creation of new community residential facilities and through harnessing practical strategies to provide space for psychosocial support services (e.g. converted cargo containers used for support groups—a strategy used for provision of variety of services in overcrowded clinics in South Africa).

*‘You know I think government should be more involved…you know supporting NGOs more financially and to establish more such [community residential] centres because even with the fees involved here there is always a waiting list. There is not enough space to accommodate everybody.’*—Service user representative 1.

## Discussion

This study was undertaken against a backdrop of low levels of service provision for community-based PSR in South Africa, particularly in rural areas [2], and limited intersectoral collaboration [3, 37]. Although local contexts and policy and resourcing environments differ, similar challenges are likely being faced in other African countries and wider LMIC contexts. South Africa is however poised to benefit from positive recent developments such as the introduction of new National Mental Health Policy and plans for roll out of specialist district mental health teams [15]. The study aimed to investigate challenges to intersectoral working between governmental and non-governmental actors for the provision of community-based PSR services and to gain perspectives from key informants on strategies for addressing challenges. Strategies identified are particularly relevant for other middle income countries with similar resourcing environments and service delivery platforms, but also for lower income countries seeking to make progress on provision of comprehensive mental health services.

The majority of participants in this study agreed that current levels of intersectoral collaboration for PSR were low, suggesting lack of progress since previous South African research describing intersectoral working at national level but not at district levels [3]. Participants identified isolated cases of intersectoral working, which were not supported by organisational structures. This type of working strategy has been identified internationally by WHO reports on intersectoral action for health which recognize that ‘small scale, local action’ gives motivated individuals opportunities to form strong, productive relationships. However dependence on individual action is not sustainable for long-term provision of country-wide services [1]. WHO case studies on intersectoral action for health suggest the need for involvement of a variety of intersectoral partners, each with support from their own organisation for involvement in the intersectoral action [38]. For example the Sonagachi HIV/AIDS International Project (SHIP) in India was based on a partnership between WHO, All India Institute of Hygiene and Public Health (AIIHPH), the British Council, and a number of Ministries and local NGOs. This project was aimed at sex workers in Kolkata, initially aiming to provide treatment and prevention of sexually transmitted infections in sex workers in the area. The involvement of partners whose work focused on outcomes beyond direct health outcomes led to broader economic empowerment of sex workers as a result of literacy and microcredit programmes and the institution of a member organisation [38]

Key challenges to intersectoral work that emerged from the data were (i) inadequate communication and structure in working relationships; (ii) the ongoing challenge of delineating roles and responsibilities; and (iii) a perceived lack of support between sectors. Although there is strong recognition in the public health sphere internationally of the need for intersectoral collaboration, even for HIC there is little peer reviewed evidence on the real-world application of this strategy [39]. Challenges identified in this study however do mirror those identified in a HIC context in which a lack of culture of ‘working together’, lack of knowledge of one sector on the work of other sectors, and lack of structures and guidelines for joint work have also been identified as particular challenges [10].

Key informants provided several feasible strategies for addressing these challenges, outlined below. These strategies correspond well with research from HIC indicating success factors for intersectoral working to be effective communication and planning at both the organisational and service-delivery levels; improving relevant professionals’ knowledge and skills; and appropriate resource allocation [10, 38, 39]. This suggests the relevance of the recommendations from this study both to South African policy makers and health programmers, but also to those in other African countries and beyond.

### Strategies identified by participants for improving intersectoral collaboration for PSR

#### Promoting sector fulfilment of roles

Participants in this study identified the need to clarify, in a practical sense, the roles of intersectoral partners in PSR programming, to ensure understanding between sectors of other sectors’ roles, and to build capacity where needed to fulfil these roles. To some extent different participants had different solutions to problems identified (e.g. increasing funding to NGOs *vs* direct employment by DOH or DOSD of more social workers). While this underscores the need for sector role clarification, it also suggests potential for actors to move beyond their previously defined roles through sharing resources and responsibilities in the intersectoral partnership.

#### Proposed DOH role

Given the recognized role (supported by most participants in this study) of DOH in leading the process of provision of PSR services, recommendations for DOH action at the service delivery level and at the organizational/planning level were made.

At the service delivery level the role of tertiary staff in diagnosis and of primary health workers in ongoing medication management was well supported by participants in this study. Recommendations for PHC nurses include improving their capacity to ‘create an informed, motivated, and adherent patient’ [26] in line with the development of the South African Health System which is embracing an integrated chronic disease management model (ICDM) [26]. PHC nurse provision of psychoeducation for those with severe mental disorders and their caregivers is the most obvious activity indicated. There is evidence that even hospital-based staff in South Africa see themselves as ill equipped to provide PSR, given the lack of focus of previous mental health policies and training on this area [34] so significant inputs for capacity building would be required. The acknowledgement of participants in this study of the need for DOH service providers to provide a lead role in provision of PSR also points to the role of the PHC nurse in ‘case management’, the need for which was emphasized. In this context the case management role would of necessity be scaled back in comparison to the HIC Intensive Case Management model [40] but would entail maintaining contact with patients, tracking adherence and hospital/specialist referrals, and making referrals to other services (e.g. social services) as required, and is in line with the ICDM call for an increased role in holistic care for PHC nurses [26]. Other developments in line with the ICDM would be additional ‘case management’ functions (e.g. working with families, health promotion, initiation of support groups) to be provided by ward-based outreach teams [26], and promotion of medication adherence and tracing of treatment defaulters for all chronic conditions by current HIV counsellors [26]. The role of primary health care workers at clinic level in providing the case management function and managing referrals at service points may be applicable to a variety of LMIC. In very low resource contexts this function may feasibly be provided by another cadre (e.g. community health workers).

At the planning/management level, the South African National Mental Health Policy framework and strategic plan has the objective of roll-out of at least one specialist mental health team per district by the end of 2015 [15] which provides a clear opportunity for progress on intersectoral working. The Terms of Reference for these specialist teams cover their role in improving referral pathways from primary care to specialist services, but do not emphasise referral to other services in other sectors [15]. This could be a key opportunity for improvement of intersectoral working at the district level. These teams would also need to include specifics for intersectoral collaboration in the development of district mental health care plans as a core objective under their terms of reference [15]. The role of referral of people with severe mental illness to primary health services, and to the social/community services that are available, is one that could be strengthened in the work of, for example, community health workers, who, as indicated, may be present even in very low resource settings in LMIC contexts.

#### Proposed DOSD role

Similar to the role of DOH in provision for biomedical aspects of treatment, the role of DOSD in provision of social grants and funding NGOs was well supported by participants in this study. Some participants in this study recommended that DOSD should take a broader approach. Since PSR is grounded in a community-based rehabilitation framework, services should not focus only on psychosocial support but also on social inclusion and equalisation of opportunities for people with psychosocial disability [41, 42]. This aligns with growing acknowledgement across LMIC of the need to dovetail approaches for mental health and social development [42], although there has been limited integration of mental health into social development in some countries’ development models to date [43]. DOSD can have a key role in incorporating service users with mental illness into their overall community development approach, specifically to address calls to alleviate the impact of poverty on those with severe mental illness [44, 45]. Increasing evidence is mounting (largely from NGOs such as BasicNeeds) on the feasibility, cost effectiveness and benefit of inclusion of those with mental disorders in community development models [46]. This approach would also support South African progress on alignment with UN proposed sustainable development goals which include a target to promote mental health and wellbeing [47]. Practically, integration across the mental health and social development sectors would be beneficial across LMICs and will be encouraged by development of cross cutting indicators for monitoring progress (e.g. mental health outcomes of social development programmes) [43].

The unavailability, described by participants in this study, of social workers to meet the needs of mental health service users underscores previous calls for national training centres for psychiatric social workers in South Africa [48]. General social workers are overburdened and focused on the needs of orphans and vulnerable children and families living in poverty—similar challenges are likely being faced particularly in African countries and those with high rates of HIV prevalence similar to South Africa. More social workers focused on psychosocial disability are greatly needed, but there are unlikely to be sufficient numbers in the near future, underscoring the need for para professionals and working in a task-sharing model. The suggestion was also made in this study to assign DOSD social workers to primary health clinics to work closely with the district mental health team. However this approach could be hampered since levels of stigma against people with severe mental illnesses, particularly schizophrenia, are high in South Africa [49]. Social workers without previous experience of working with mental health care users may need training and support to reduce stigmatising behaviour.

DOSD participants in this study had somewhat limited knowledge of the South African Mental Health Care Act and the National Mental Health Policy, and of psychosocial disability in general, which may have contributed to the perspective that addressing this is a ‘health’ issue. This is likely a challenge in other relevant sectors (e.g. housing) [3]. Mutual training between sectors may be beneficial for intersectoral working [39] in South Africa and other LMIC contexts particularly to work towards reduction of stigma against mental health care users.

#### Improving communication, structured working relationships and leadership

Communication challenges were identified between the majority of participants in the study, both at the level of individuals mental health providers (e.g. between health workers and social workers) and between different levels (provincial, district) of the health and social service systems. Clarifying and supporting pathways for communication and referral between levels of the health system is a recognised priority for the planned district mental health teams in South Africa [15] but work on pathways with DOSD and other community services needs to be similarly emphasized. Several participants also identified the existing social development clusters as the key avenue for potential communication on issues relating to PSR services. A vital part of the district mental health team planning/management role as identified earlier could be in the representation of issues relating to PSR in this forum as well as a promoting the institution of cluster meetings if these do not exist. Supporting this in the South African context is the documented need across the sectors for increasing capacity at provincial level for managers to conduct operational planning in an integrated way [37]. Other LMICs may have similar fora in place and it is possible that similar challenges hinder their functionality. Where possible these fora should be harnessed and strengthened for the promotion of PSR. In settings where they do not exist, their set up would represent a key step towards promoting intersectoral working for PSR and other health and social development issues. A directive from national and provincial levels of the social development cluster (or its equivalent in other LMIC) for requirements for well-functioning clusters with regular meetings and monitored actions would increase accountability for intersectoral work.

This study highlighted that at the provincial and district level, service level agreements between intersectoral partners are not present, but would be beneficial. A key challenge for many countries is the focus on limited mandates for government departments and the fact that each department has its own specific ‘language and culture’—which leads to people working in ‘silos’ and competing for resources [1], which was mentioned by several participants in this study. A written agreement with roles and responsibilities for service providers to assemble a comprehensive PSR service at district level, with negotiated and agreed input from all sectors, is a feasible approach to address this challenge in South Africa and elsewhere. Key performance indicators on intersectoral action for service providers and managers in the sectors may also be relevant.

In terms of leadership, the main area of need seems to be clear directives from DOH for different sectors concerning implementation of PSR services from the national to provincial level [50] and beyond this to district level. Leadership for intersectoral collaboration by Health sectors across LMIC at national and provincial levels will require building trust and enabling other sectors to focus on the broader benefits to society of intersectoral action (e.g. social justice and equity) [1] as well as showing clearly why intersectoral action is appropriate for provision of this service [38]. These in turn should help to make mental health relevant to other sectors and enable pooled resources to flow in the required direction. Governments as a whole also need to foster a more positive orientation to intersectoral action as part of their ‘fundamental stewardship responsibility in health’ [1] [51]. This means a recognition from high levels of leadership that this way of working needs to be built into structures and working practices, and therefore requires a dedicated budget (e.g. for monitoring frameworks for intersectoral action) [1].

#### Direction of available resources to community-based PSR services

Budget constraints for mental health services are an ongoing challenge, particularly considering the burden of other chronic and communicable diseases in South Africa [6]. This is borne out by the lack of a specific budget for mental health at district level and at the subdistrict level where services are provided. There is consequently a lack of a budget line specifically for PSR, as highlighted by several participants in this study. With respect to budgets for mental health, the constraints experienced in South Africa (and other LMIC) are unlikely to change in the near future as identified by a DOH national representative in this study. Participants in this study did however highlight the contrast between the lack of provision of PSR services with the substantially more developed substance abuse rehabilitation programme, which has a dedicated funding stream from DOSD, and in which a functional partnership between DOH and DOSD has been instituted. Experience from a variety of countries shows that the wider resource constraints in the public sector, as well as administrative structures, act as an impediment to intersectoral collaboration [51] and that long-term sustainable intersectoral action can be costly and time intensive [1]. The most discussed need for resource allocation by participants in the study was for the provision of infrastructure and management of community residential facilities which is addressed in the National Mental Health Policy. There were however contrasting perspectives on the availability of resources for PSR services between district and national level participants. District level NGO and DOH representatives felt the effects of scarce resources on the ground and a sense of competition within DOH for different disease priorities affecting the country. This is the manifestation of the public sector resource-constrained environment which fosters competition instead of collaboration between sectors [9] in many LMIC. By contrast, national representatives generally felt resources for PSR were available. This reflects knowledge that resources at tertiary level are available, but there remains limited redirection of these resources to community level. There is a crucial need for accountability and assessment of the adequate transfer of resources in this direction [52] but reallocation of resources to community services is a complex undertaking for South Africa and other LMIC. There are key learnings from recent progress in Brazil involving negotiation with municipalities for resource reallocation from hospital beds to community mental health services (through Centers of Psychosocial Care—CAPS), residential services, cash transfers and psychosocial support for community integration as well as programmes for employment/income generation for people with mental disorders [53]. Sixty-six percent of the Brazilian population was estimated to be covered adequately through CAPS services as of 2010. These positive developments have been grounded on ‘political will, adequate financial resources and attention to technical aspects of the implementation’ [54]. South Africa and other LMIC will need to bolster each of these to see progress on intersectoral provision of community mental health services. Working with municipalities as a key intersectoral partner was not identified as theme in this study, although one district level DOH participant did highlight the role municipalities could have in provision of community residential facilities. The National Mental Health Policy does state the role of local government in providing for transport, housing and recreational needs of people with mental disabilities [15] but the practical involvement of municipalities as a key partner for intersectoral provision of PSR in South Africa will be an important area for future investigation.

## Limitations

A limitation of this study was the focus on perspectives from DOH informants with fewer participants being from DOSD, NGOs and service user organisations. Some representatives had previous working relationships with the research group which may have led to a desirability response. The interview schedule also did not ask specifically for success stories so it may not have specifically captured positive perceptions related to intersectoral collaboration.

## Future research

As a potential strategy for effective provision of PSR services, observational studies showing how intersectoral work can be implemented in practice [39] will be valuable. These will allow documentation of good practices and guidelines for intersectoral working. An example in the South African context could be the national substance abuse programme as identified by participants in the study. Observational and evaluative studies of intersectoral working practices would also allow for creation of platforms for knowledge sharing from HIC where intersectoral work may be more well-developed to LMIC, and between LMIC where similar challenges may be faced.

## Conclusion

The continuing lack of focus on PSR points to ongoing marginalization of people with severe mental illness and blockage of progress in provision of required services, as has been the case in various African countries [14]. Intersectoral provision of PSR services in South Africa has emerged in this study as a complex challenge due to resource allocation and scarcity, inadequate organizational structures, and lack of trust, communication and clarity on roles. These challenges are clearly substantial, and help to explain lack of progress in the area despite widespread acknowledgement of the importance of working in this way. This study has outlined several directions for progress to address these challenges. How these are addressed will hinge on far-sighted political will and leadership to provide for services for this group of service users, particularly since intersectoral work may only show results in the long term [1]. On the other hand, greater cohesion between the health and social development spheres may help to provide the momentum and resources needed for appropriately scaling up services for mental health care users [43].

## Abbreviations

AIIHP: All India Institute of Hygiene and Public Health
BREC: Biomedical Research Ethics Committee
CAPS: Centers of Psychosocial Care
COREQ: Consolidated Criteria for Reporting Qualitative Research
DOH: Department of Health
DOSD: Department of Social Development
HIC: High Income Country
ICDM: Integrated Chronic Disease Management
LMIC: Low and Middle Income Country
NGO: Non-Governmental Organisation
PRIME: Programme for Improving Mental Health Care
PSR: Psychosocial Rehabilitation
SASSA: South African Social Services Agency
SHIP: Sonagachi HIV/AIDS International Project
